# Postoperative prosthetic mitral valve occlusion due to left atrial thrombus during veno-arterial extracorporeal membrane oxygenation: a case report

**DOI:** 10.1186/s40981-022-00586-5

**Published:** 2022-12-06

**Authors:** Naoto Kiuchi, Yusuke Seino, Mai Yamamoto, Seidai Katagiri, Shunichi Takagi, Takeshi Nomura, Takahiro Suzuki

**Affiliations:** 1 Department of Anesthesiology, Nihon University School of Medicine, 30-1 Kamicho Oyaguchi, Itabashi-ku, Tokyo, Japan; 2 Department of Intensive Care Medicine, Tokyo Women’s Medical University, 8-1 Kawada-cho, Shinjuku-ku, Tokyo, Japan; 3 Department of Anesthesiology, St. Marianna University School of Medicine, 2-16-1 Sugao, Miyamae-ku, Kawasaki, Japan

**Keywords:** Prosthetic valve, Mitral valve occlusion, Left atrial thrombus, Extracorporeal membrane oxygenation, Transesophageal echocardiography

## Abstract

**Background:**

Anticoagulation using heparin is generally used to prevent thrombus formation during mechanical circulatory support, such as veno-arterial extracorporeal membrane oxygenation (VA-ECMO). However, during the early period following cardiac surgery, anticoagulation becomes more difficult due to the greater risk of critical bleeding complications.

**Case presentation:**

A 71-year-old man presented with acute prosthetic valve occlusion caused by left atrial thrombus formation and bioprosthetic valve thrombosis during peripheral VA-ECMO following mitral valve replacement (MVR) despite continuous heparin administration and loading of antiplatelet agents. The VA-ECMO flow rate decreased 10 h after the intensive care unit (ICU) admission after MVR. Exploratory transesophageal echocardiography (TEE) examination revealed a left atrial thrombus, prosthetic valve obstruction by the thrombus, and an intrapericardial hematoma.

**Conclusions:**

Intracardiac thrombus formation might occur during VA-ECMO despite appropriate anticoagulation and loading of antiplatelet agents. Exploratory TEE examination was helpful in the detection of intra-atrial thrombus formation after cardiac surgery and surgical decision-making.

**Supplementary Information:**

The online version contains supplementary material available at 10.1186/s40981-022-00586-5.

## Background

Anticoagulation with heparin is generally used to prevent thrombus formation during mechanical circulatory support (MCS), such as veno-arterial extracorporeal membrane oxygenation (VA-ECMO). VA-ECMO patients after cardiac surgery, however, require attention regarding appropriate anticoagulation because of concerns about bleeding complications. A previous meta-analysis reported that 42.9% of VA-ECMO patients who underwent cardiac surgery had bleeding complications that required re-operation to achieve hemostasis [1]. We present here a case of acute prosthetic valve occlusion due to a left atrial thrombus and bioprosthetic valve thrombosis during peripheral VA-ECMO following mitral valve replacement (MVR) despite continuous heparin administration and loading of antiplatelet agents. Exploratory transesophageal echocardiography (TEE) in the intensive care unit (ICU) was helpful in diagnosis and surgical decision-making.

## Case presentation

**Fig. 1:**
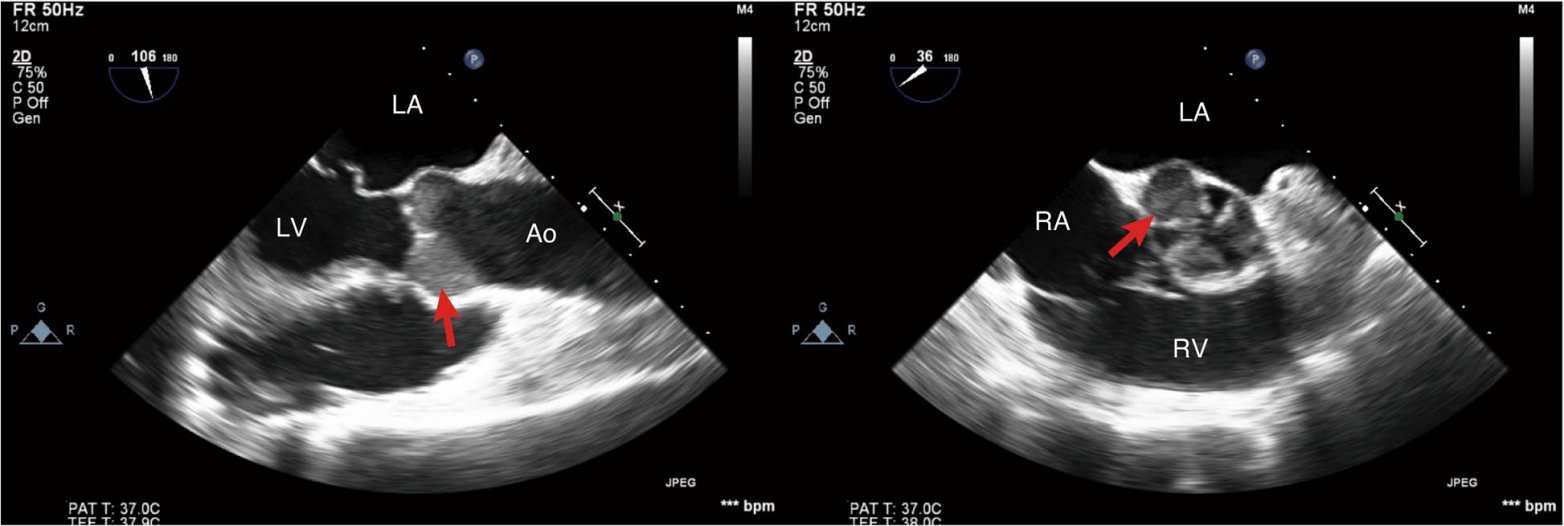
Mid-esophageal aortic valve long axis (left) and short axis (right) views. Spontaneous echo contrast (arrow) was observed in the sinus of Valsalva after the induction of general anesthesia for the initial mitral valve replacement surgery. Ao, ascending aorta; LA, left atrium; LV, left ventricle; RA, right atrium; RV, right ventricle

A 71-year-old man was transported to our hospital with acute myocardial infarction due to total occlusion of the obtuse marginal branch of the circumflex coronary artery. After prasugrel and aspirin loading, percutaneous coronary intervention (PCI) under intra-aortic balloon pumping (IABP) was performed. Since circulatory collapse and ventricular fibrillation occurred after reperfusion of the coronary artery, mechanical ventilation with tracheal intubation was commenced and VA-ECMO was established via the left femoral artery to the left common iliac artery and right femoral vein to right atrium cannulation. The flow rate of VA-ECMO at the start was 3.8 L/min. Using transthoracic echocardiography (TTE), mitral regurgitation (MR) due to papillary muscle rupture and moderate tricuspid regurgitation was diagnosed and emergency MVR using a bioprosthetic valve was planned 5 h after PCI on the same day. Intraoperative TEE before the surgical procedures showed severe mitral regurgitation due to a flail anterior leaflet, spontaneous echo contrast in the sinus of Valsalva, slight movement of the aortic valve cusps, and diffuse severe hypokinesis of the left ventricle (LV) (Fig. 1, Additional file 1 Video 1). The preoperative activated clotting time (ACT) was 234 s, and was prolonged to 534 s after administration of 10,000 units of heparin (200 units/kg). Cardiopulmonary bypass (CPB) was established with ascending aortic cannulation and venous drainage via the superior vena cava and right femoral vein. MVR with a bioprosthetic valve (Epic 27 mm, St. Jude Medical, MN, USA) and tricuspid annuloplasty (Tailor Band 29 mm, St. Jude Medical) were performed. At the time of weaning from CPB, TEE showed diffuse akinesis of LV, no thrombus in the left atrium, and disappearance of the spontaneous echo contrast in the sinus of Valsalva.

**Fig. 2:**
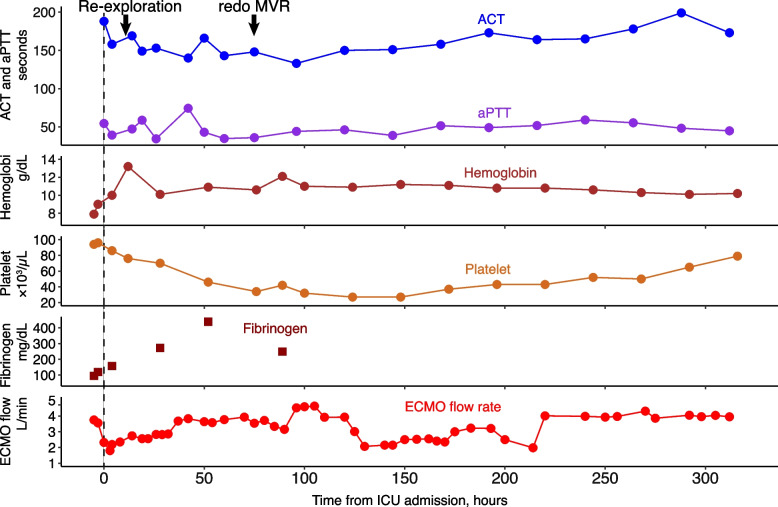
The changes in activated clotting time, activated partial thromboplastin time, extracorporeal membrane oxygenation flow rate, hemoglobin, platelet, and fibrinogen levels after the ICU admission. ACT, activated clotting time; aPTT, activated partial thromboplastin time; ECMO, extracorporeal membrane oxygenation; MVR, mitral valve replacement

**Fig. 3:**
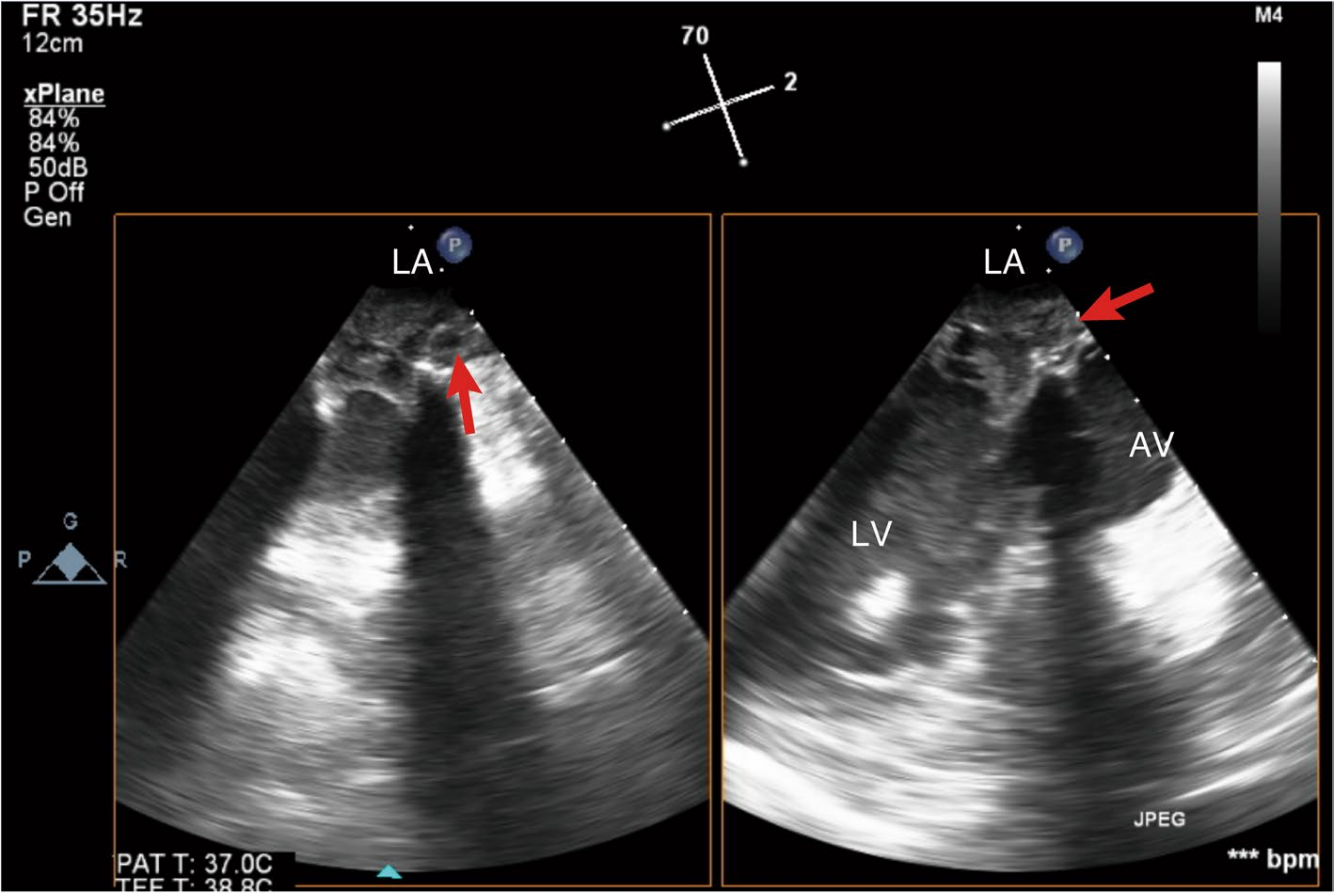
Mid-esophageal mitral commissural view (left) and long axis view (right). The prosthetic mitral valve occluded by a left atrial thrombus (arrow) was observed on postoperative day 1, after the initial mitral valve replacement by transesophageal echocardiography performed in the ICU. AV, aortic valve; LA, left atrium; LV, left ventricle

However, since the patient could not be weaned from CPB because of severe ventricular dysfunction, VA-ECMO via the left femoral artery and right femoral vein cannulation was established again with the concomitant use of IABP with 1:1 support, and he was transferred to the ICU. There was no effective pulse pressure after weaning from CPB. Before leaving the operating room, 50 mg of protamine was administered via a peripheral vein, resulting in decrease of ACT to 211 s. In the ICU, heparin was continuously administered at about 2000 units/hour, measuring ACT and activated partial thromboplastin time (aPTT) with targeting an ACT of 140–180 s due to concerns about the risk of bleeding (Fig. 2), and the VA-ECMO flow rate was adjusted to 3 L/min/m^2^. However, the measured VA-ECMO flow rate decreased to 1.3–1.7 L/min/m^2^ after 10 h of admission to the ICU, and blood transfusion was required to maintain the VA-ECMO flow rate at 2 L/min/m^2^. Thus, exploratory TEE was performed in the ICU to determine the cause of the reduced VA-ECMO flow rate on postoperative day (POD) 1. Unexpectedly, TEE revealed a left atrial thrombus extending to the ostium of the right superior pulmonary vein, obstruction of the prosthetic valve by the thrombus (Fig. 3, Additional file 2 Video 2), and right ventricular collapse due to an intrapericardial hematoma, and no opening of the aortic valve. Considering the patient’s critical status, the surgeon decided to perform only re-exploration for intrapericardial hematoma removal on POD 1. Mechanical circulatory support was continued to maintain systemic perfusion to prevent and improve organ dysfunction. As a result, the patient's general status improved, including a return to sinus rhythm, improvement in left ventricular wall motion, and increased urinary output.

**Fig. 4:**
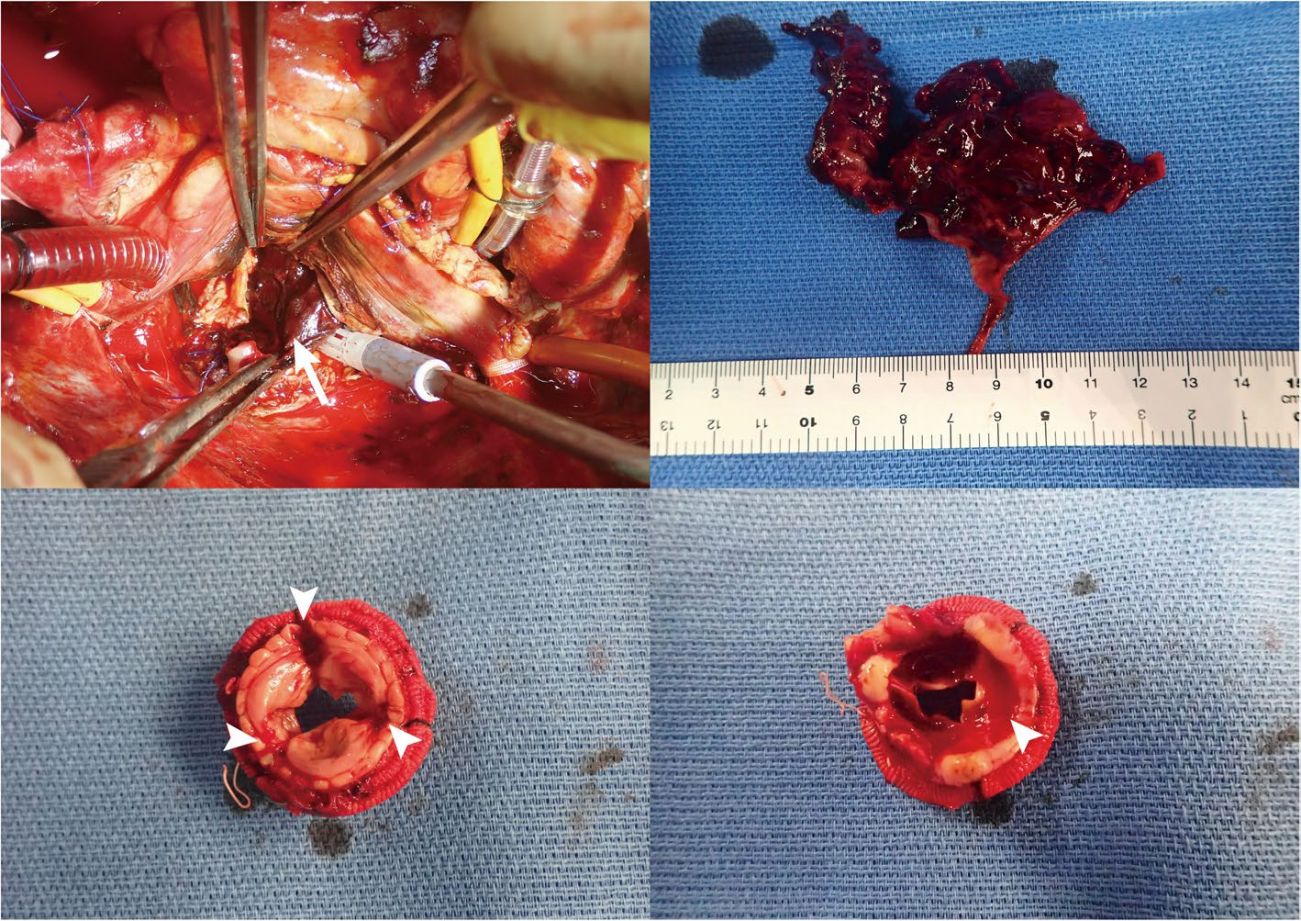
The operative field during re-mitral valve replacement. There was a blood clot stuck (arrow) in the mitral valve (upper left). The upper right image shows the extracted thrombus, and the lower left and right images show the removed prosthetic mitral valve (the left atrial and ventricular sides, respectively) with blood clots (arrow heads)

**Fig. 5:**
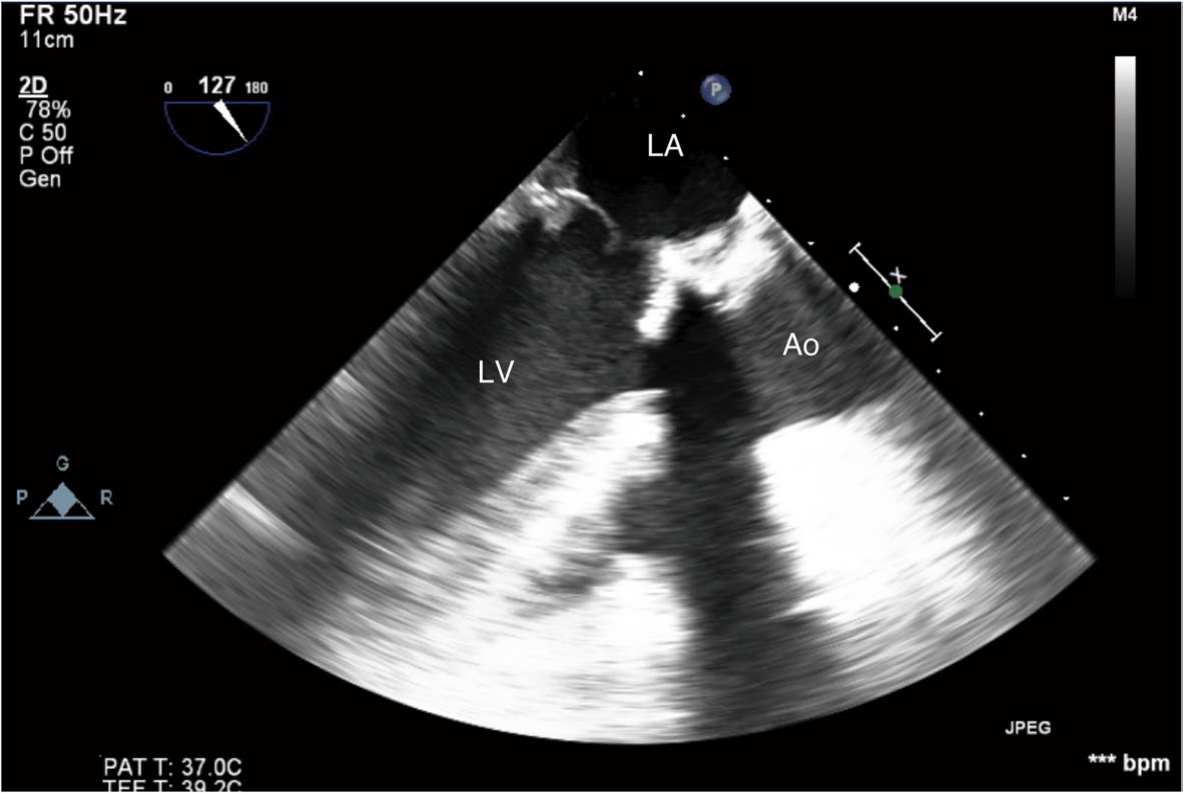
Mid-esophageal long axis view in the ICU on postoperative day 8. There was no evidence of thrombus formation. Ao, ascending aorta; LA, left atrium; LV, left ventricle

Subsequently, on POD 4, the patient underwent left atrial thrombectomy and re-MVR with a bioprosthetic valve (Epic 27 mm, St. Jude Medical). Blood clots adhered to the bioprosthetic valve and were diagnosed as prosthetic valve thrombosis (Fig. 4). Intraoperative TEE showed the left atrium filled with thrombus at the start of the operation, with no thrombus in the cardiac chambers after the re-MVR (Fig. 5, Additional file 3 Video 3). The patient was returned to the ICU under central VA-ECMO support with ascending aortic cannulation and right femoral vein drainage and was finally weaned from central VA-ECMO on POD 15 without ECMO flow problems and IABP with 1:2 support on POD 16 after the initial surgery. Tracheostomy was performed on POD 27 because of postoperative pneumonia and ICU-acquired respiratory muscle dysfunction. After improvement of the patient’s respiratory status, he was discharged from the ICU on POD 39, discharged from the hospital without any neurological complications on POD 50, and transferred to a rehabilitation hospital. Tracheostomy tube was removed on POD 86.

## Discussion

We identified two important clinical issues from this case. Postoperative prosthetic mitral valve occlusion due to left atrial thrombus and bioprosthetic valve thrombosis could occur during VA-ECMO despite appropriate systemic anticoagulation and loading of antiplatelet agents. Exploratory TEE in the ICU was useful for the detection of unexpected intracardiac thrombus formation and surgical decision-making.

While bioprosthetic valve thrombosis, including in the chronic phase, was reported to have occurred in 11.6% of patients who underwent bioprosthetic valve replacement, early thrombosis around the bioprosthetic mitral valve is a rare phenomenon [2]. Although bleeding has been emphasized as a complication during MCS after cardiac surgery, a retrospective single-center study reported that 10% of patients with prosthetic valves developed prosthetic valve thrombosis during VA-ECMO for post-cardiotomy shock [3, 4]. The risk factors for bioprosthetic valve thrombosis include extremely low cardiac output, low transvalvular flow, left atrial dilatation, atrial fibrillation, and hypercoagulability [5]. On the other hand, there are several factors related to intracardiac thrombus formation with VA-ECMO, including altered circulatory flow, vascular injury, and altered blood coagulability [6]. In particular, low cardiac output and low transvalvular flow as a consequence of VA-ECMO can lead to thrombus formation in the left heart chambers [7, 8]. The LV afterload increases due to retrograde blood flow from peripheral VA-ECMO with femoral artery cannulation and femoral vein drainage; thus, in cases with low cardiac output, intracardiac blood stasis is likely to occur, promoting thrombus formation [9]. In the present case, the patient’s cardiac output was extremely low because of frequent potentially fatal arrhythmias and prolonged cardiogenic shock. Spontaneous echo contrast distal to the aortic valve in intraoperative TEE in the initial MVR indicated the presence of blood stasis in the sinus of Valsalva. Additionally, the patient’s cardiac function deteriorated further after surgery, resulting in additional stasis in the left atrium and ventricle, as well as in the ascending aorta, which probably led to the development of a thrombus that occluded the prosthetic mitral valve.

The fundamental approach to preventing thrombus formation during MCS is reportedly anticoagulant therapy and ventricular unloading for the prevention of LV distention [10]. Since thrombus formation can occur in patients with extremely low cardiac function despite appropriate systemic anticoagulation, early LV unloading for the prevention of blood stasis is critical to preventing thrombus formation [10]. In the present case, the extreme LV dysfunction contributed to blood stasis and thrombus formation, because LV failed to resist retrograde blood flow from the femoral artery cannula and could not generate an adequate cardiac output after the initial MVR. By contrast, thrombus formation did not occur after the re-MVR. Improved cardiac function and antegrade blood flow of the central ECMO from the ascending aorta cannula might have led to unloading of LV and reduction of blood stasis after the re-MVR [3, 6, 9]. These differences from the first surgery might have contributed to the prevention of thrombus formation. LV unloading techniques such as Impella or LV venting cannula were not used, given the risk of vascular complications and bleeding and our institution's limited experience with their use in the perioperative period [11].

Monitoring of anticoagulation therapy for VA-ECMO differs from facilities. ACT, aPTT, and the anti-factor Xa assay can be used for monitoring anticoagulation with heparin. In the present case, ACT and aPTT were measured. The target ACT of 180 to 220 s is recommended for anticoagulation of VA-ECMO [12]. However, because of concerns about the risk of bleeding, the target ACT was set at 140 to 180 in the present case. There is little evidence regarding anticoagulation during ECMO in such complicated situations as in the present case. A systematic review of ECMO cases managed without anticoagulation reported that the incidence of thrombosis was comparable with or without anticoagulation and severe bleeding occurred even in 28% of ECMO patients without anticoagulation [13]. In addition, there are some reports that the management with low ACT or without anticoagulation was beneficial in patients with a high risk of bleeding [14, 15]. Given these results, the anticoagulation strategy at a lower ACT value than recommended in the ELSO guidelines would be acceptable in patients at high risk of bleeding.

Exploratory TEE for hemodynamic instability in the ICU played a pivotal role in the detection of intracardiac thrombus formation and surgical decision-making. Vigilant monitoring with echocardiography is important in diagnosing thrombus formation in the prosthetic valve or cardiac chambers and in determining treatment strategy [8, 16]. In most previously reported cases, intracardiac thrombus during VA-ECMO was diagnosed using TEE [6]. TEE has certain advantages in the diagnosis of intracardiac thrombus after cardiac surgery, because obtaining images of sufficient quality for the diagnosis of thrombus formation is sometimes difficult with transthoracic echocardiography due to the narrow acoustic windows and artifacts [17]. In post-cardiac surgery patients with severe cardiac dysfunction requiring MCS and at risk for thrombus formation, such as the present case, early and vigilant monitoring with TEE might facilitate the diagnosis of thrombus formation and early therapeutic intervention [18, 19].

In conclusion, thrombus formation could occur during VA-ECMO after cardiac surgery despite appropriate anticoagulation and loading of antiplatelet agents. Exploratory TEE in the ICU was helpful for the detection of thrombus formation and surgical decision-making in such cases. In post-cardiac surgery patients with severe cardiac dysfunction requiring VA-ECMO, TEE in the ICU might improve patient outcomes by facilitating therapeutic intervention.

## Supplementary Information

**Additional file 1:** **Video 1.**

**Additional file 2:** **Video 2.**

**Additional file 3:** **Video 3.**

## Data Availability

The data in this case report are available from the corresponding author on reasonable request.

## Abbreviations

ACT: Activated clotting time
aPTT: Activated partial thromboplastin time
CPB: Cardiopulmonary bypass
ICU: Intensive care unit
IABP: Intra-aortic balloon pumping
LV: Left ventricle
MCS: Mechanical circulatory support
MR: Mitral regurgitation
MVR: Mitral valve replacement
PCI: Percutaneous coronary intervention
POD: Postoperative day
TEE: Transesophageal echocardiography
TTE: Transthoracic echocardiography
VA-ECMO: Veno-arterial extracorporeal membrane oxygenation
